# Case of Abscess of the Liver, Communicating with, and Discharging through the Right Lung

**Published:** 1840-03-14

**Authors:** John I. Sinnickson

**Affiliations:** Brazoria, Texas


### CASE OF ABSCESS OF THE LIVER,

*communicating with, and discharging through the Right Lung.* By John I. Sinnickson, M. D.

In giving a history of the following case, I am under the necessity of making numerous extracts from letters written by Mr. C. K. Reese, nephew of the afflicted gentleman, whose intelligence enabled him to give a correct, though not a minute statement of the course of the disease. The patient lived fifteen miles from Brazoria, my place of residence, which rendered it impossible for me to witness the progress of the disorder, and consequently unable to communicate an accurate detail of symptoms, which must cause the perusal of the case to be unsatisfactory.

Abram Bowman, aged 53, of a strumous habit, and by occupation a planter, states, that when about four years of age, he had a white swelling on the right side of the thorax, in the region of the fifth or sixth rib; through the carelessness of the nurse in letting him fall, one of the ribs was detached and came away; he gradually recovered, and enjoyed, as he states, excellent health until the period of the last war in the United States, when he was enlisted for the army in Kentucky. Whilst in the service, he was again attacked with a disease of the same character, and in the same place; it was punctured by a surgeon, and about one quart of pus discharged therefrom. After this, he only recovered his health in such a degree, as to enable him to pursue his avocation; and upon exposure was always very susceptible to cold, accompanied with severe cough. About the first of March last, I received a letter requesting my advice for Mr. B., who was complaining of a severe pain on the right side of the thorax, with cough, in consequence of his being exposed in a storm, and getting very wet. Thinking it might probably be pleurisy, I advised venesection, if there was much fever or pain, until the latter was relieved, or an effect produced upon the pulse; if to the contrary, to abstain from it, and administer laxatives in connection with diaphoretics, confinement, spare diet, and blisters to be applied after the inflammatory action was in a degree subdued. On the 15th inst. Mr. Reese communicated to me as follows: "When I wrote to you about the health of my uncle, I was of opinion that he had the pleurisy, as he was harassed with a cough, which has increased; I have no doubt but what it is a pulmonary disease of some kind; the cough troubles him very much, in the night especially, and causes him to expectorate a large quantity of phlegm, which I have not discovered as yet to be streaked with blood, and which, I believe, is generally regarded as a symptom of phthisis. The pain in the side continues not so bad, and I think he has no fever. The treatment you recommended, was adopted with much relief, and I am under the impression if something could be administered to relieve the cough, he would do well." Counter-irritation with antimony ointment, laxatives, diaphoretics, pectoral mixture, spare diet, &c., were recommended. In the course of a fortnight, Mr. Reese called upon me, and I took the opportunity to interrogate him minutely in regard to the symptoms his uncle complained of, and was convinced at the time, that Mr. B.'s disease was phthisis pulmonalis, and expressed my opinion as such to Mr. Reese.

I would mention that there were no symptoms through which it could be ascertained that hepatitis, or any other disease of the liver, was then existing; on account, as is presumed, of the pulmonic irritation being so great as to render them obscure, except it was pain extending down to the right hypochondriac region, and thence darting to the inferior dorsal vertebræ. I recommended, however, the course of treatment necessary to palliate the symptoms, and requested Mr. R. to inform me by every opportunity of the progress of the disease. In a few days I received a letter, stating that the pain had become very severe along the course, as before stated, cough exceedingly harassing, the matter discharged assuming a yellowish appearance; heat in the palms of the hands and soles of the feet very distressing; fever, bowels rather loose, occasional night sweats, but not profuse, and the disease presenting such a serious aspect that the patient was becoming despondent, and desirous of seeing me.

The following day, the 2d of April, I visited Mr. B., and while examining him in regard to the symptoms, he directed my attention to the right lumbar region, contiguous to the floating ribs, as being the principal seat of the pain, where I discovered a slight, circumscribed, indurated swelling, which led me to a more thorough investigation, and to pronounce the disease what I believe Dr. Wilson Philip terms "hepatic phthisis." At this period, there were no symptoms indicative of suppuration in the part; the swelling was indurated; he complained not of the pain being of a throbbing character, nor did he complain of rigours, or any great sensation of weight or heaviness in the region of the liver; and the above symptoms were attributable to phthisis. I was of the opinion that there was an abscess then forming in the liver, and expressed it to his friends, mentioning at the time, that the preservation of his life depended solely upon the course it should take to discharge itself. Poultices were directed to be applied to the swelling, with a view of promoting its pointing externally; laxatives, &c., to palliate symptoms; nourishing diet, with bark and wine, to support his strength, as there was much debility, accompanied with great emaciation.

This course was pursued with but little variation, until the 22d of April, when I again visited him, and upon the way met with my friend, Dr. John M'N. Stewart, whose scientific attainments I highly appreciated, and resolved in this case to avail myself of; at my solicitation, he accompanied me to Mr. B.'s residence. Upon examining the swelling, fluctuation of matter was distinctly perceptible, and every symptom of an abscess ascertained. In the mean time, the patient informed us that while in a recumbent posture, about three hours previous to our arrival, he plainly felt something rising from the seat of the tumour, through the right lung, and immediately he discharged from his mouth about half a pint of yellowish matter, which, from its greatly impeding respiration, caused him to assume an erect position, when he felt it flowing back to the swelling. We punctured it with an abscess lancet, and discharged therefrom about five pints of healthy yellow pus. It is worthy of observation, that during the evacuation of pus, a distinct crepitus was perceptible to the hand, upon being placed over the abscess, and air issued from the orifice with a hissing noise; proving incontrovertibly, that the abscess communicated with the lung. He bore the operation without any symptoms of exhaustion, and expressed himself greatly relieved; he emerged from a state of despair, became very cheerful, and asserted that he thought he should soon become a well man. A poultice was directed to be applied to the orifice, and the pus permitted to be discharged every four or five hours, as much as possible; wine, bark, and elixir of vitriol with a nourishing diet was recommended, with the pediluvium of nitro-muriatic acid and water. I received information a few days afterwards, that the orifice continued open, and more than a pint of pus had been discharged; mind cheerful, appetite good, and able to sit up almost constantly throughout the day; since which, intelligence has been received at various periods, that he is rapidly recovering, capable of using much exercise, and accumulating strength and flesh rapidly. About three weeks since, Mr. B. visited this town, appearing in better health than I had ever seen him. He stated that the wound was completely healed, the cough had left him, and his general health was better than it had been for several years.

*Brazoria, Texas, Aug.* 4, 1838.

